# Identifying key genes related to inflammasome in severe COVID-19 patients based on a joint model with random forest and artificial neural network

**DOI:** 10.3389/fcimb.2023.1139998

**Published:** 2023-04-11

**Authors:** Haiya Ou, Yaohua Fan, Xiaoxuan Guo, Zizhao Lao, Meiling Zhu, Geng Li, Lijun Zhao

**Affiliations:** ^1^ Department of Gastroenterology, Shenzhen Bao'an Traditional Chinese Medicine Hospital, Guangzhou University of Chinese Medicine, Shenzhen, China; ^2^ Traditional Chinese Medicine Innovation Research Center, Shenzhen Hospital of Integrated Traditional Chinese and Western Medicine, Shenzhen, China; ^3^ Laboratory Animal Center, Guangzhou University of Chinese Medicine, Guangzhou, China

**Keywords:** COVID-19, severe, lung, inflammasome, random forest, artificial neural network

## Abstract

**Background:**

The coronavirus disease 2019 (COVID-19) has been spreading astonishingly and caused catastrophic losses worldwide. The high mortality of severe COVID-19 patients is an serious problem that needs to be solved urgently. However, the biomarkers and fundamental pathological mechanisms of severe COVID-19 are poorly understood. The aims of this study was to explore key genes related to inflammasome in severe COVID-19 and their potential molecular mechanisms using random forest and artificial neural network modeling.

**Methods:**

Differentially expressed genes (DEGs) in severe COVID-19 were screened from GSE151764 and GSE183533 *via* comprehensive transcriptome Meta-analysis. Protein-protein interaction (PPI) networks and functional analyses were conducted to identify molecular mechanisms related to DEGs or DEGs associated with inflammasome (IADEGs), respectively. Five the most important IADEGs in severe COVID-19 were explored using random forest. Then, we put these five IADEGs into an artificial neural network to construct a novel diagnostic model for severe COVID-19 and verified its diagnostic efficacy in GSE205099.

**Results:**

Using combining *P* value < 0.05, we obtained 192 DEGs, 40 of which are IADEGs. The GO enrichment analysis results indicated that 192 DEGs were mainly involved in T cell activation, MHC protein complex and immune receptor activity. The KEGG enrichment analysis results indicated that 192 GEGs were mainly involved in Th17 cell differentiation, IL-17 signaling pathway, mTOR signaling pathway and NOD-like receptor signaling pathway. In addition, the top GO terms of 40 IADEGs were involved in T cell activation, immune response-activating signal transduction, external side of plasma membrane and phosphatase binding. The KEGG enrichment analysis results indicated that IADEGs were mainly involved in FoxO signaling pathway, Toll-like receptor, JAK-STAT signaling pathway and Apoptosis. Then, five important IADEGs (AXL, MKI67, CDKN3, BCL2 and PTGS2) for severe COVID-19 were screened by random forest analysis. By building an artificial neural network model, we found that the AUC values of 5 important IADEGs were 0.972 and 0.844 in the train group (GSE151764 and GSE183533) and test group (GSE205099), respectively.

**Conclusion:**

The five genes related to inflammasome, including AXL, MKI67, CDKN3, BCL2 and PTGS2, are important for severe COVID-19 patients, and these molecules are related to the activation of NLRP3 inflammasome. Furthermore, AXL, MKI67, CDKN3, BCL2 and PTGS2 as a marker combination could be used as potential markers to identify severe COVID-19 patients.

## 1 Introduction

The coronavirus disease 2019 (COVID-19), a disease caused by SARS-CoV-2, has been spreading astonishingly and caused catastrophic losses worldwide. Since the outbreak of the epidemic, COVID-19 has caused more than 6.6 million deaths worldwide. Even if the mortality of Omicron decreases, the high mortality of severe COVID-19 patients is still a problem that needs to be solved urgently (Fano-Sizgorich et al., 2022).

Respiratory failure is one of the main causes of death in severe COVID-19 patients (Ohuabunwa et al., 2022). Meanwhile, studies found that patients with severe COVID-19 had severe respiratory symptoms during hospitalization, including lower oxygen saturation, and diminished lung function compared to those after non-severe COVID-19 (Bieńkowski et al., 2022; Lenoir et al., 2022). Compared to patients with non-severe coronavirus pneumonia, lower oxygen saturation and lung function in the first year in patients with severe COVID-19. In addition, inappropriate hyperinflammatory response caused by excessive activation of inflammasome is an important pathological manifestation in severe COVID-19 patients (Tay et al., 2020; Milara et al., 2022). Increasing evidence also indicated that inflammasomes is a multiprotein signaling platforms that assemble in the cytosol in response to microbial infections and cell stress and plays an important role in the occurrence and development of severe COVID-19 (Baena Carstens et al., 2022). Respiratory inflammasome activation and dysregulation of the NACHT, leucine-rich repeat, and pyrin domain-containing protein 3 (NLRP3) inflammasome in circulating neutrophils are associated with worsening of COVID-19 (Jeong et al., 2022; Leal et al., 2022). The activation and dysregulation of the NACHT, leucine-rich repeat, and pyrin domain-containing protein 3 (NLRP3) inflammasome in circulating neutrophils are associated with worsening of COVID-19. Notably, inflammasome genetic variants are associated with protection to clinical severity of COVID-19 (de Sá et al., 2022). Furthermore, an elegant longitudinal study performed in patients with COVID-19 found a correlation between the late-stage pathology in COVID-19 and cytokines linked to the inflammasome pathway, including IL-1β and IL-18 (Lucas et al., 2020).

Although, the interaction between SAES-CoV-2 and angiotensin-converting enzyme 2 (ACE2) could induce microglia to produce a large number of NLRP3 inflammasome (Albornoz et al., 2022; Potere et al., 2022). The mechanism of inflammasome activation accelerating the occurrence and development of severe COVID-19 is still unclear. Therefore, our study would use a joint model with random forest and artificial neural network to identify key genes related to inflammasome in severe COVID-19 patients. Random forest can be applied to approximate functions and dynamics by learning from samples, and it is a powerful deep learning and artificial intelligence tools which is used to predict key disease biomarkers widely. Based on the important genes of random forest prediction, we further build neural networks to evaluate the accuracy of these important genes in disease prediction. It is helpful to clarify the relevant mechanism of SARS-CoV-2 damage to lung tissue and provide new targets and new ideas for new drug research and development.

## 2 Materials and methods

### 2.1 Data collection and data processing

**Figure 1:**
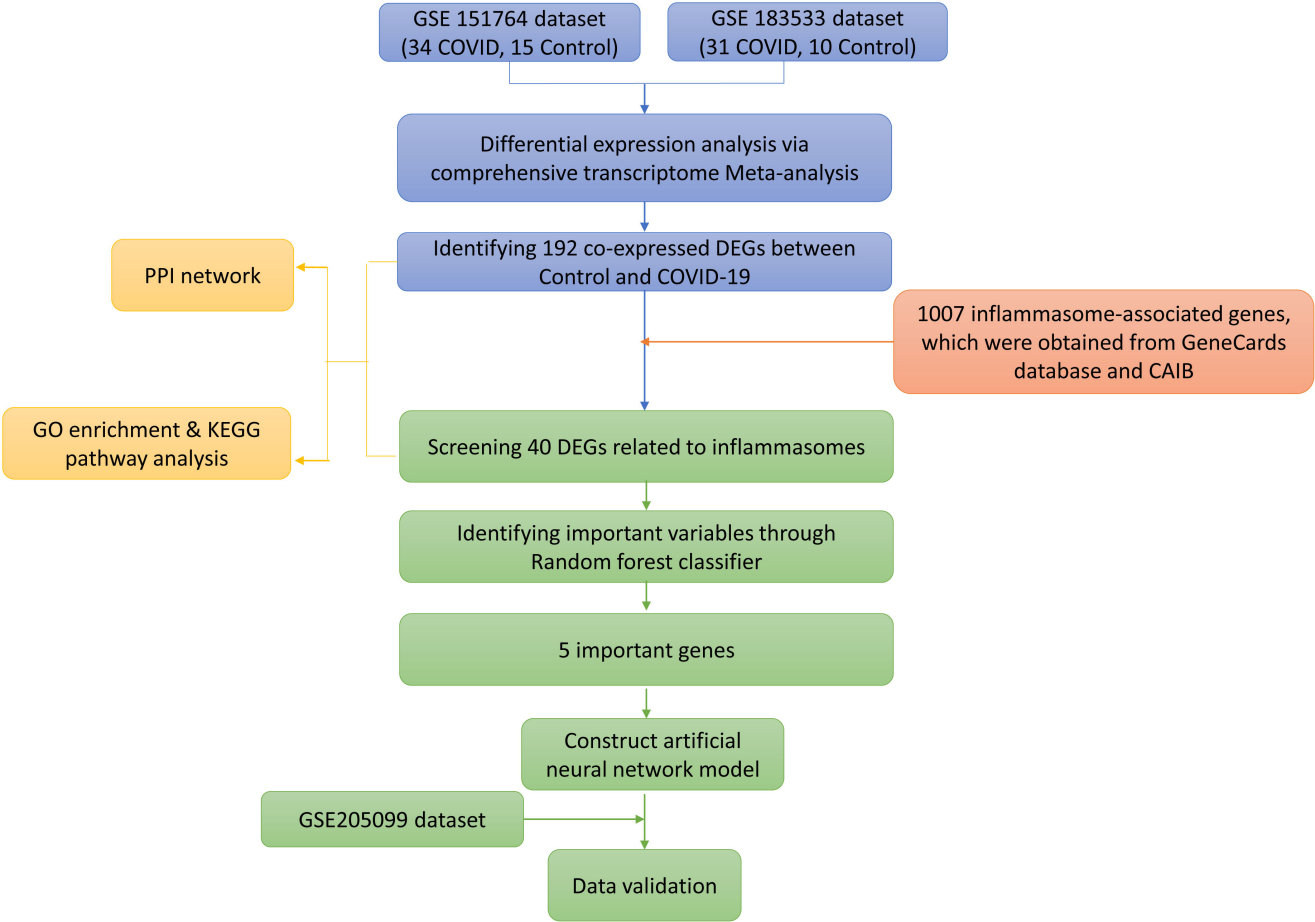
Study flowchart.

Data analysis procedures of this study are shown in **Figure 1**. The original expressing profile data of GSE151764 (16 deceased COVID-19 patients, 6 patients who died from non-infectious causes), GSE183533 (31 deceased COVID-19 patients, 10 patients who died from non-infectious causes) and GSE205099 (12 deceased COVID-19 patients, 4 patients who died from non-infectious causes, each biological replicate has 4 technical replicates) were downloaded from NCBI-GEO (http://www.ncbi.nlm.nih.gov/geo). The related annotation information, such as the platforms, the probes, and ID conversion, was obtained from the GEO database. When multiple probes corresponded to one gene or microRNA symbol, the highest expression level of multiple probes was used as the expression level of the corresponding gene. R (v4.1.3), Bioconductor (v3.14), org.Hs.eg.db (v3.14.0) were used for ID conversion.

### 2.2 Batch effect adjustment and statistical meta-analysis

The pre-processed and normalized data sets were subjected to the well-established ComBat procedures to reduce potential study-specific batch effects (Johnson et al., 2007). Then, we used the principal component analysis (PCA) to examine the results visually. The meta-analysis was performed using the web-based tool—NetworkAnalyst (Zhou et al., 2019). Stouffer’s method (based on inverse normal transformation) incorporates weight (i.e. based on sample sizes) into the calculation. Combining *P* value (*P*< 0.05) was used to select differentially expressed genes (DEGs) during the datasets of GSE151764 and GSE183533. Clustering heatmap analysis of differentially expressed genes was done in the NetworkAnalyst.

### 2.3 Selection of inflammasome-associated genes

Inflammasome-associated genes were obtained from GeneCards database (https://www.genecards.org/) and cancer analysis of inflammasome balance (CAIB, http://l.neuroscience.org.cn/).

### 2.4 Protein-protein interaction network construction and analysis

Protein-protein interaction (PPI) information of DEGs was acquired and established *via* the search tool for retrieval of interacting genes (STRING) database (v11.5). The standard of significant interaction was set as a combined score ≥ 0.7. We also established a PPI association network related to inflammasome through STRING database.

### 2.5 Gene ontology and Kyoto Encyclopedia of genes and genomes pathway enrichment analysis

To explore the biological significance of DEGs and inflammasome-associated DEGs, gene ontology (GO) and Kyoto Encyclopedia of genes and genomes (KEGG) pathway enrichment analyses were performed *via* the R package “clusterProfiler” (v4.2.2) to identify significantly enriched GO terms (threshold of q-value < 0.05) and significantly enriched KEGG pathways (threshold of q-value < 0.05).

### 2.6 Screening key inflammasome-associated DEGs with the random forest model

We used the R package “randomForest” (v4.7-1) to construct a random forest model to screen key inflammasome-associated DEGs. The number of random seeds and decision trees was set as 10000 respectively in the original random forest classifier. The Gini coefficient method was used to obtain the dimensional importance value of all variables from the constructed random forest model. Screening out the top 5 inflammasome-associated DEGs as important genes in severe COVID-19 patients for subsequent model construction and validation. The R package “pheatmap” (v1.0.12) was used to perform clustering analysis of the screened key genes for the heatmap in this dataset.

### 2.7 The construction and verification of artificial neural network model

We used GSE151764 and GSE183533 dataset as the training set for the construction of the artificial neural network model. The R package “neuralnet” (v1.44.2) was used to construct an artificial neural network model of those important genes. Then, four hidden layers were set as the model parameter to construct a classification model of severe COVID-19 patients through the predicted gene weight information. Then we used GSE205099 dataset as the test set to verify the accuracy of the model. The R packages “pROC” (v1.18.0) and “ggplot2” (v3.3.5) were used to calculate the verification results of AUC classification performance and draw the ROC curve.

## 3 Results

### 3.1 Differential expression analysis *via* comprehensive transcriptome meta-analysis

**Figure 2:**
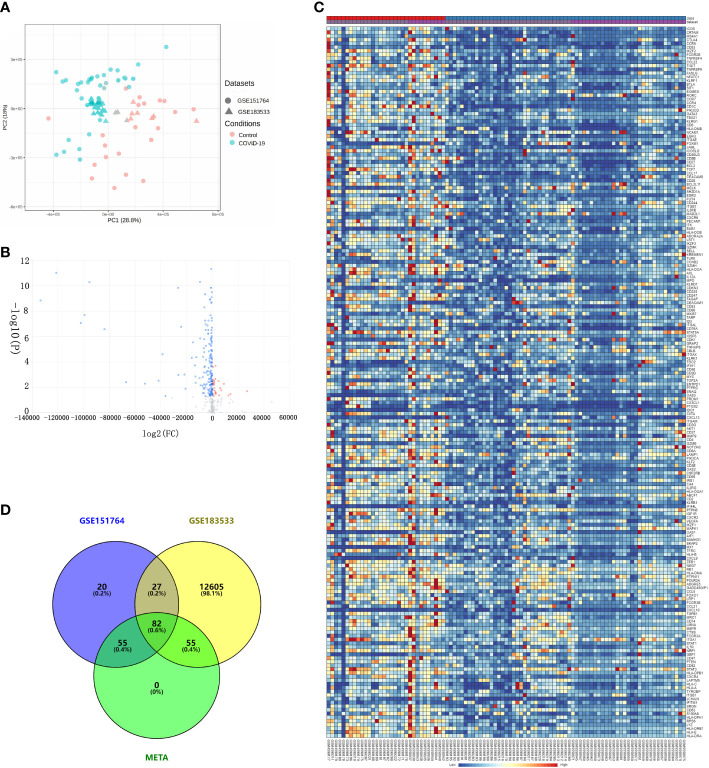
Differential expression analysis *via* comprehensive transcriptome meta-analysis using the GSE151764 and GSE183533 datasets. **(A)** Bidimensional principal component analysis for COVID-19 and control samples; red, control group; blue, COVID-19. **(B)** Volcano plot showing the results of differential expression analysis between control and COVID-19 groups. **(C)** Heatmaps of DEGs co-expressed between the COVID-19 and control groups. Legend at the top right indicates log-fold change in gene expression level. Horizontal axis, each sample; vertical axis, each gene. Blue and red colors represent low and high expression values, respectively. **(D)** Venn diagram of DEGs.

Before downstream analysis, all samples in GSE151764 and GSE183533 were assessed by two Principal Component Analysis (PCA). The results showed that samples of severe COVID-19 patients were distinctly distinguished from normal samples (**Figure 2A**).

Using combining *P* value < 0.05, we obtained 192 differentially expressed genes (DEGs) (**Figures 2B, C**). Then, the hierarchical heatmap shows that these 192 DEGs can be clustered into two distinctive groups, one of the group consists of 27 genes that are upregulated in patients with severe COVID-19, and the other group of 165 genes are down-regulated (**Figure 2D** and **Table S1**).

### 3.2 PPI network construction and enrichment analyses

**Figure 3:**
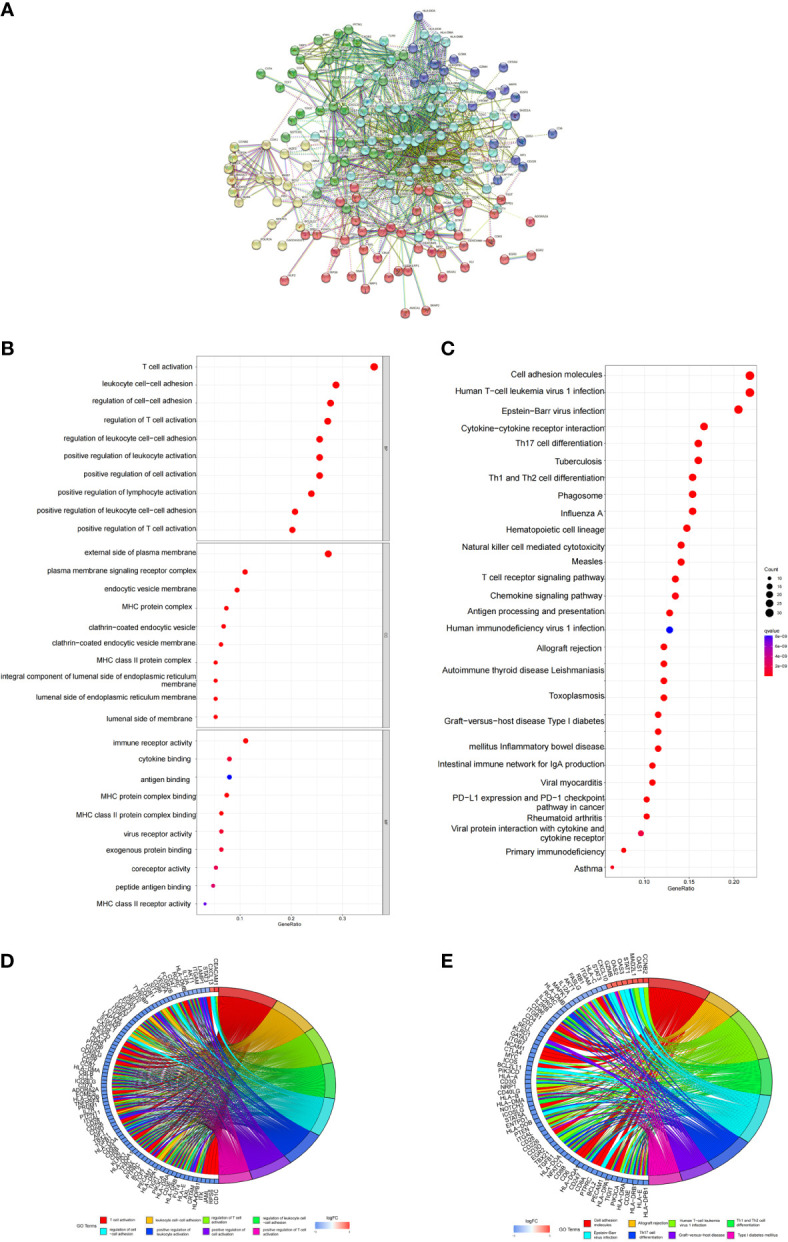
PPI networks of DEGs, and functional analysis. **(A)** PPI network of DEGs. Disconnected nodes are hidden. **(B, C)** The top GO items enriched for DEGs. **(D, E)** KEGG pathway enrichment results for DEGs.

In order to explore the biological significance of these DEGs in the pathogenesis of COVID-19, we first constructed a PPI network using STRING database. *Via* the STRING website, we found the PPI network of predicted genes consisted of 180 nodes and 1217 edges (Disconnected nodes were hidden) (**Figure 3A**). According to the kmeans clustering method, the PPI network can be divided into five clusters (**Table S2**). Among them, 50 genes such as FOXO1, ADORA2A and AXL are taken as the first cluster; BCL2, CDK1, RB1 and other 17 genes as the second cluster; CCL17, MAPK1 and other 33 genes as the third cluster; B7RP1, IL7R and 60 genes as the fourth cluster; AIF1, CD226 and other 22 genes as the fifth cluster.

Then, enrichment analysis of GO and KEGG pathways were carried out through R software package. We obtained 1393 Biological Process (BP), 65 Cellular Component (CC) and 83 Molecular Function (MF) by enriching 192 DEGs (**Table S3**). The top 10 GO terms of genes with significantly different expression levels were visualized in the GO bubble chart. The GO enrichment analysis results indicated that these significant DEGs were mainly involved in T cell activation, MHC protein complex and immune receptor activity (**Figures 3B, D**). For KEGG pathway enrichment analysis, we got the top 125 terms of genes by q-value<0.05 (**Table S4**). The KEGG enrichment analysis results indicated that these GEGs were mainly involved in Th17 cell differentiation, IL-17 signaling pathway, mTOR signaling pathway and NOD-like receptor signaling pathway (**Figures 3C, E**).

**Figure 4:**
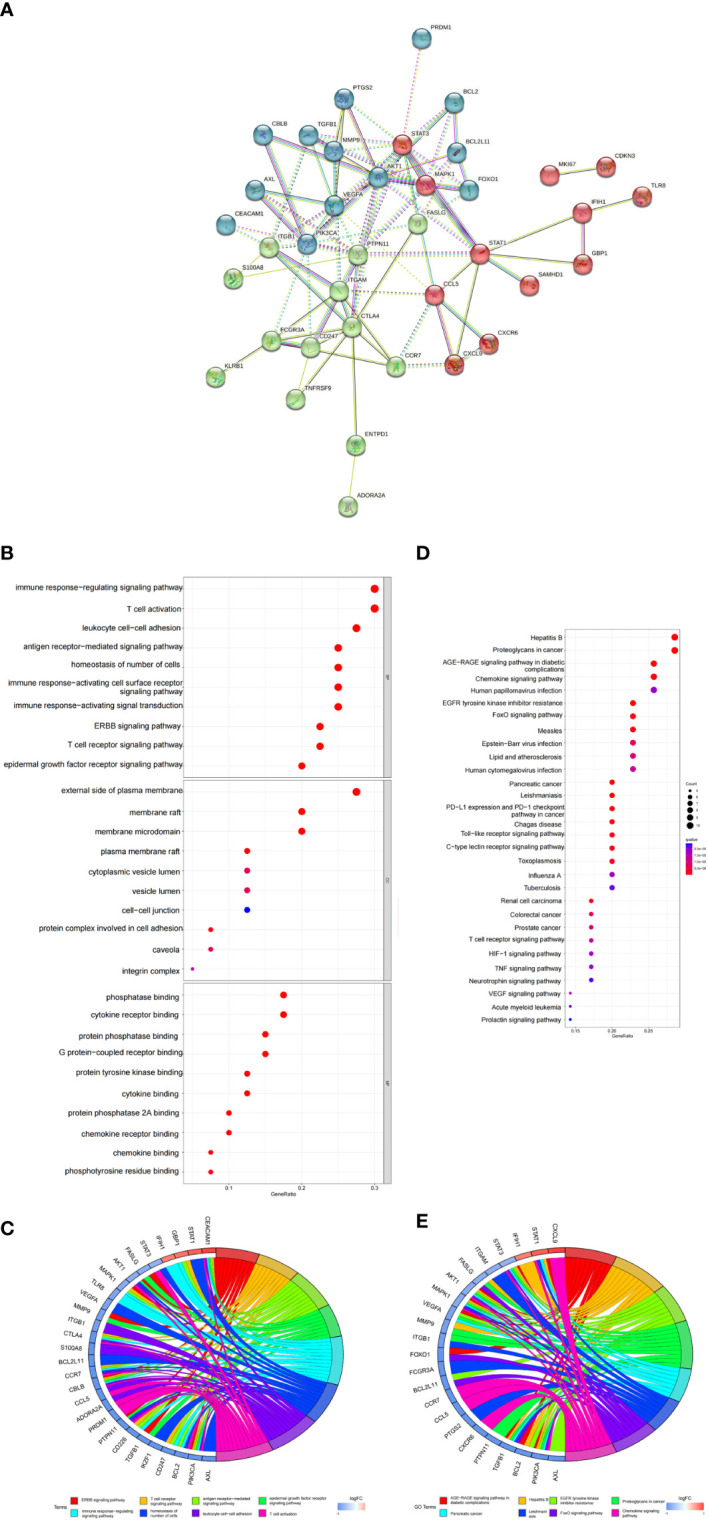
PPI networks of IADEGs, and functional analysis. **(A)** PPI network of IADEGs. Disconnected nodes are hidden. **(B, C)** The top GO items enriched for IADEGs. **(D, E)** KEGG pathway enrichment results for IADEGs.

In order to further explore the biological function of DEGs related to inflammasomes (IADEGs), 40 IADEGs were screened from 1007 inflammasome-associated genes, which were obtained from GeneCards database and CAIB (**Table S5**). The PPI network of inflammasome-associated DEGs consisted of 38 nodes and 94 edges, accompanied with average node degree 4.7 (Disconnected nodes were hidden) (**Figure 4A**). Then, we obtained 1446 BP, 23 CC and 39 MF by enriching 40 IADEGs (**Table S6**). The top GO terms were involved in T cell activation, immune response-activating signal transduction, external side of plasma membrane and phosphatase binding (**Figures 4B, C**). The KEGG enrichment analysis results indicated that these IADEGs were mainly involved in 122 KEGG signaling pathway, including FoxO signaling pathway, Toll-like receptor, JAK-STAT signaling pathway and Apoptosis (**Figures 4D, E** and **Table S7**).

### 3.3 Constructing the random forest model to screen key genes related to inflammasome in severe COVID-19 patients

**Figure 5:**
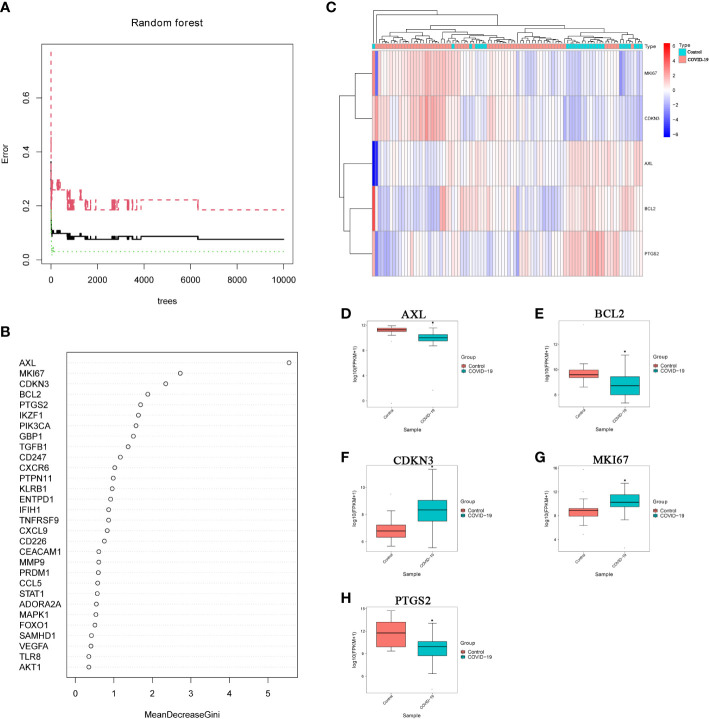
Screening important IADEGs using a random forest model. **(A)** Relationship between the number of decision trees and model error; x-axis, number of decision trees; y-axis, error rate of the constructed model. **(B)** Importance of all variables in the random forest classifier determined using the Gini coefficient method; x-axis, mean decrease of the Gini index; y-axis, variables. **(C)** Heatmap of k-means clustering for the GSE151764 and GSE183533 dataset. Colors in the graph from red to blue indicate the change from high to low expression levels. In the upper part of the heatmap, the blue band indicates control samples and the red band COVID-19 samples. **(D–H)** Differential expression of AXL, MKI67, CDKN3, BCL2 and PTGS2 the GSE151764 and GSE183533 datasets. *P<0.05, vs Control group.

We selected 6193 trees as the parameter of the random forest model, which represented a stable error in the model through referring to the relationship between the model error and the number of decision trees (**Figure 5A**). In the modeling process, we used the Gini coefficient method to measure the importance of each variable according to decreased mean square error and model accuracy (**Figure 5B**). Then, we obtained 5 IADEGs (AXL, MKI67, CDKN3, BCL2 and PTGS2), which with a mean decrease of Gini index was the top 5, as important variables for subsequent analysis. Based on these 5 variables, we performed the k-means clustering of the dataset, which suggested that these 5 IADEGs could be used to distinguish the sample of severe COVID-19 patients from the normal samples (**Figure 5C**). Among, AXL, BCL2 and PTGS2 were clustered as a group with high expression in the normal sample and low expression in the COVID-19 sample. On the contrary, CDKN3 and MKI67 were clustered as another group with low expression in the normal sample and high expression in the COVID-19 sample (**Figures 5D–H**).

### 3.4 The construction of the artificial neural network model and the evaluation of ROC cure

**Figure 6:**
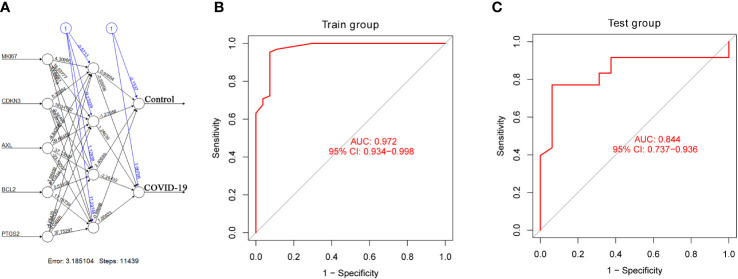
Construction and verification of an artificial neural network model. **(A)** Visualization of the artificial neural network model. **(B, C)** ROC curve evaluation of the train and test groups.

According to the output results of the neural network model, it is illuminated that the entire training was performed in 11439 steps. Among the output results, the predicted weights of each hidden neuron layer were 0.90554, -1.27058, 2.40695 and -0.98668 (**Figure 6A**). Next, we drew the ROC curve to evaluate the predicted performance; the AUC value of 5 important IADEGs was 0.972 (**Figure 6B**). The AUC value of the ROC curve was verified as 0.844 by analysis of lung samples from the GSE205099 dataset (**Figure 6C**). Therefore, AXL, MKI67, CDKN3, BCL2 and PTGS2 might be key markers for occurrence and development of severe COVID-19.

## 4 Discussion

The SARS-Cov-2 strain are continuously mutating and increasing infection prevalence rapidly. In the meanwhile, the number of deaths due to infection is also increasing. Although there are several vaccines and anti-viral drugs, they cannot effectively curb the number of deaths caused by COVID-19 (Zhang et al., 2020). Developing potential therapies requires understanding the molecular basis for disease and the pathological mechanism of the main lesion sites, especially the lungs (Arunachalam et al., 2020). Moreover, severe COVID-19 can lead to fatalities through a hyperinflammatory cytokine storm. It also is associated with high levels of pro-inflammatory cytokines and an influx of blood-derived myeloid populations into the lung (Channappanavar and Perlman, 2017; Liao et al., 2020; Silvin et al., 2020). In response to infection mediated by CD16 and ACE2 receptors, human macrophages activate inflammasomes, release IL-1 and IL-18 and undergo pyroptosis, which leads to the hyperinflammatory state of the lungs (Broz and Dixit, 2016; Sefik et al., 2022). Moreover, tissue-resident macrophages, but not epithelial and endothelial cells,have activated inflammasomes in lung autopsies of patients with COVID-19 (Junqueira et al., 2022). Also, inflammasome activation correlated with dysfunctional T cells and low monocytic TMEM176B expression in severe COVID-19 (Duhalde Vega et al., 2022). However, targeting the inflammasome in severe COVID-19 may be a potential strategy, this pathway may have a protective role, as its loss increases morality to other respiratory viruses (Allen et al., 2009; Ichinohe et al., 2009; Thomas et al., 2009; Yap et al., 2020). Therefore, identifying key disease genes and pathways related to inflammasomes will provide a foundation for the development of drug targets and biomarkers (Jin et al., 2022).

Here, we used bioinformatics and systems biology methods to clarify the molecular regulatory mechanism of inflammasomes in severe COVID-19. We used two COVID-19 lung tissue datasets (GSE151764 and GSE183533) from the GEO database as the training set for analyzing DEGs between COVID-19 and control samples. The KEGG enrichment analysis results indicated that these 192 GEGs were mainly involved in Th17 cell differentiation, IL-17 signaling pathway, mTOR signaling pathway and NOD-like receptor signaling pathway. Among, Th17 cell differentiation might be associated with NLRP3 inflammasomes (Liang et al., 2021). Also, IL-1 or IL-18 synergizes with IL-23, can promote IL-17-production from Th17 cells and γδ T cells, and this process can be regulated by autophagy (Mills et al., 2013). Both NOD-like receptor signaling pathway and mTOR signaling pathway are closely related to the activation of inflammasomes (Li et al., 2018; Xu et al., 2022). Also, the KEGG enrichment analysis results of 40 IADEGs showed similar pathways, including Th17 cell differentiation, mTOR signaling pathway and NOD-like receptor signaling pathway. These results demonstrate that inflammasomes play a potential important role in severe COVID-19.

Inflammasome formation include a two-step process. Firstly, it is usually provided by cytokines or Toll-like receptor (TLR) agonists, and primes a cell for inflammasome activation by upregulating inflammasome-related genes and inducing post-translational modifications to component proteins. It is usually provided by cytokines or Toll-like receptor (TLR) agonists and initiates inflammasome activation in cells by up-regulating inflammasome-related genes and inducing post-translational modifications of component proteins. During infection, viral nucleic acids or surface proteins may activate TLRs to supply this signal. Then, a cytosolic signal nucleates the inflammasome through activation of one of many sensors, including NLRP3, and absent in melanoma 2 (AIM) (Broz and Dixit, 2016; Zheng et al., 2021). Afterwards, we further used the random forest model to screen key IADEGs. Based on the random forest model, we obtained 5 IADEGs (AXL, MKI67, CDKN3, BCL2 and PTGS2) which are the highest importance. At present, there is little literature directly proving the effect of the above five genes on the inflammasomes of COVID-19. However, we could analyze the effect of these five genes on inflammasomes in severe COVID-19 by combining existing literature. Thereinto, AXL encode a member of the Tyro3-Axl-Mer (TAM) receptor tyrosine kinase subfamily, which not only plays a key role in inhibition of TLR-mediated innate immune response, but also participates in EGFR tyrosine kinase inhibitor resistance pathway (O'Bryan et al., 1991; D'Arcangelo et al., 2002). Furthermore, the activation of NLRP3 inflammasomes increased when the expression of AXL decreased (Han et al., 2016). Our results also show that AXL is decreased in the lung tissue of patients with severe COVID-19, which suggests that there is an overactivation of inflammasomes. Meanwhile, existing research has not found that the differentially expression of AXL in healthy controls and patients with mild COVID-19. Nevertheless, recently studies showed the interaction between AXL and the SARS-CoV-2 S glycoprotein on the host cell paved the way for the entry of the virus into the host cell (Naik et al., 2022; Zdżalik-Bielecka et al., 2022). Bemcentinib and Gilteritinib, pharmacological inhibitions of AXL, showed promising as potential COVID-19 therapy (Boytz et al., 2022; Naik et al., 2022; Zdżalik-Bielecka et al., 2022). We inferred that inhibition of AXL could prevent the SARS-CoV-2 from entering the host cell, but it can promote the over activation of inflammatory factors in lung tissue to cause more serious inflammatory reaction. Therefore, these inhibitors cannot be popularized in clinical practice.

Interestingly, previous study also showed MKI67 could be a novel biomarker signature for SARS-CoV-2 pathogenesis (Lawal et al., 2022). Also, MKI67 and BCL2 as a marker combination that enables the tracking of nascent memory cells within the effector phase (Crauste et al., 2017). MKI67 and BCL2 act as a marker combination and are able to track nascent memory cells within the effector phase. The strength and magnitude of antibody and memory B cells induced following SARS-CoV-2 infection depend on the severity of the disease (Akhtar et al., 2022). Moreover, NF-ĸB signaling pathway is a critical anti-apoptotic or survival route mediated by SARS-CoV-2 (Yapasert et al., 2021). Such NF-ĸB signaling pathway promotes viral survival, proliferation, and inflammation by inducing the expression of apoptosis inhibitors, especially BCL2. Moreover, BCL2 also participates in HIF-1 signaling pathway, p53 signaling pathway, PI3K-Akt signaling pathway, NOD-like receptor signaling pathway, JAK-STAT signaling pathway and other signal pathways to affect cell survival. At the same time, BCL2 in the peripheral blood of COVID-19 patients was negatively correlated with the severity of the disease (Lorente et al., 2021).

In addition, when the expression level of BCL2 and PTGS2 decrease, NLRP3 inflammasomes will be hyperactivated, which leads to apoptosis (Ding et al., 2018; Iskusnykh et al., 2021). Meanwhile, PTGS2 also involves several signal pathways related to inflammasomes, such as NF-ĸB signaling pathway, IL-17 signaling pathway and TNF signaling pathway. However, there is little research on how CDKN3 regulates inflammasomes, compared with AXL, MKI67, BCL2 and PTGS2. At present, scholars speculate that it may play a role in cell cycle regulation, so further research is needed (Gyuris et al., 1993; Hannon et al., 1994). Finally, our results suggest that these key genes are mainly related to NLRP3 inflammasome in severe COVID-19 patients.

Although our study advances understanding of the pathological mechanisms underlying severe COVID-19, it has several limitations. First, we cannot analyze the DEGs of severe patients with different basic diseases, due to the lack of clinical data. Second, we found that there was no significant change in the genes related to inflammasomes at the transcriptional level. It may be that key genes promote inflammatory response mainly by influencing translation or modification or aggregation of NLRP3 inflammasome related proteins. Third, some of our results are contrary to the existing understanding, so further research are needed to validate.

## 5 Conclusion

By integrating the results of random forest, we found that the five genes related to inflammasome, including AXL, MKI67, CDKN3, BCL2 and PTGS2, are important for severe COVID-19 patients, and these molecules are related to the activation of NLRP3 inflammasome. Furthermore, AXL, MKI67, CDKN3, BCL2 and PTGS2 as a marker combination could be used as potential markers to identify severe COVID-19 patients. Overall, our study provides potential new insight into the pathogenesis of inflammasome in COVID-19 and identifies crucial biomarkers as possible diagnostic and therapeutic targets.

## Data availability statement

The datasets presented in this study can be found in online repositories. The names of the repository/repositories and accession number (s) can be found in the article/**Supplementary Material**.

## Author contributions

Conceptualization, GL. Methodology, YF. Software, HO. Validation, YF. Formal analysis, YF. Writing—original draft preparation, HO and XG. Writing—review and editing, ZL and MZ. Visualization, LZ. Supervision, GL. Funding acquisition, GL and MZ. All authors have read and agreed to the published version of the manuscript.

## References

[B1] Akhtar M. Basher S. R. Nizam N. N. Kamruzzaman M. Khaton F. Banna H. A.. (2022). Longevity of memory b cells and antibodies, as well as the polarization of effector memory helper T cells, are associated with disease severity in patients with COVID-19 in Bangladesh. Front. Immunol. 13. doi: 10.3389/fimmu.2022.1052374 PMC9791541 36578502

[B2] Albornoz E. A. Amarilla A. A. Modhiran N. Parker S. Li X. X. Wijesundara D. K.. (2022). SARS-CoV-2 drives NLRP3 inflammasome activation in human microglia through spike protein. Mol. Psychiatry. Advance online publication. doi: 10.1038/s41380-022-01831-0 PMC10615762 36316366

[B3] Allen I. C. Scull M. A. Moore C. B. Holl E. K. McElvania-TeKippe E. Taxman D. J.. (2009). The NLRP3 inflammasome mediates *in vivo* innate immunity to influenza a virus through recognition of viral RNA. Immunity 30 (4), 556–565. doi: 10.1016/j.immuni.2009.02.005 19362020 PMC2803103

[B4] Arunachalam P. S. Wimmers F. Mok C. K. P. Perera R. Scott M. Hagan T.. (2020). Systems biological assessment of immunity to mild versus severe COVID-19 infection in humans. Science 369 (6508), 1210–1220. doi: 10.1126/science.abc6261 32788292 PMC7665312

[B5] Baena Carstens L. Campos D'amico R. Fernandes de Moura K. Morais de Castro E. Centenaro F. Silva Barbosa G.. (2022). Lung inflammasome activation in SARS-CoV-2 post-mortem biopsies. Int. J. Mol. Sci. 23 (21), 13033. doi: 10.3390/ijms232113033 36361818 PMC9659061

[B6] Bieńkowski C. Kowalska J. D. Paciorek M. Wasilewski P. Uliczny P. Garbacz-Łagożna E.. (2022). The clinical course and outcomes of patients hospitalized due to COVID-19 during three pandemic waves in Poland: A single center observational study. J. Clin. Med. 11 (24), 7386. doi: 10.3390/jcm11247386 36556002 PMC9787021

[B7] Boytz R. Słabicki M. Ramaswamy S. Patten J. J. Zou C. Meng C.. (2022). Anti-SARS-CoV-2 activity of targeted kinase inhibitors: Repurposing clinically available drugs for COVID-19 therapy. J. Med. Virol. 95 (1), e28157. doi: 10.1002/jmv.28157 36117402 PMC9538324

[B8] Broz P. Dixit V. M. (2016). Inflammasomes: Mechanism of assembly, regulation and signalling. Nat. Rev. Immunol. 16 (7), 407–420. doi: 10.1038/nri.2016.58 27291964

[B9] Channappanavar R. Perlman S. (2017). Pathogenic human coronavirus infections: Causes and consequences of cytokine storm and immunopathology. Semin. Immunopathol. 39 (5), 529–539. doi: 10.1007/s00281-017-0629-x 28466096 PMC7079893

[B10] Crauste F. Mafille J. Boucinha L. Djebali S. Gandrillon O. Marvel J.. (2017). Identification of nascent memory CD8 T cells and modeling of their ontogeny. Cell Syst. 4 (3), 306–317.e304. doi: 10.1016/j.cels.2017.01.014 28237797

[B11] D'Arcangelo D. Gaetano C. Capogrossi M. C. (2002). Acidification prevents endothelial cell apoptosis by axl activation. Circ. Res. 91 (7), e4–12. doi: 10.1161/01.res.0000036753.50601.e9 12364394

[B12] de Sá N. B. R. Neira-Goulart M. Ribeiro-Alves M. Perazzo H. Geraldo K. M. Ribeiro M. P. D.. (2022). Inflammasome genetic variants are associated with protection to clinical severity of COVID-19 among patients from Rio de Janeiro, Brazil. BioMed. Res. Int. 2022, 9082455. doi: 10.1155/2022/9082455 36105941 PMC9467712

[B13] Ding H. G. Deng Y. Y. Yang R. Q. Wang Q. S. Jiang W. Q. Han Y. L.. (2018). Hypercapnia induces IL-1β overproduction *via* activation of NLRP3 inflammasome: Implication in cognitive impairment in hypoxemic adult rats. J. Neuroinflamm. 15 (1), 4. doi: 10.1186/s12974-017-1051-y PMC5755461 29304864

[B14] Duhalde Vega M. Olivera D. Gastão Davanzo G. Bertullo M. Noya V. Fabiano de Souza G.. (2022). PD-1/PD-L1 blockade abrogates a dysfunctional innate-adaptive immune axis in critical β-coronavirus disease. Sci. Adv. 8 (38), eabn6545. doi: 10.1126/sciadv.abn6545 36129987 PMC9491709

[B15] Fano-Sizgorich D. Vasquez-Velasquez C. Orellana L. R. Ponce-Torres C. Gamboa-Serpa H. Alvarez-Huambachano K.. (2022). RISK OF DEATH, HOSPITALIZATION AND ICU ADMISSION BY SARS-CoV-2 VARIANTS IN PERU, a RETROSPECTIVE STUDY. Int. J. Infect. Dis. 127, 144–149. doi: 10.1016/j.ijid.2022.12.020 36563957 PMC9763211

[B16] Gyuris J. Golemis E. Chertkov H. Brent R. (1993). Cdi1, a human G1 and s phase protein phosphatase that associates with Cdk2. Cell 75 (4), 791–803. doi: 10.1016/0092-8674(93)90498-f 8242750

[B17] Han J. Bae J. Choi C. Y. Choi S. P. Kang H. S. Jo E. K.. (2016). Autophagy induced by AXL receptor tyrosine kinase alleviates acute liver injury *via* inhibition of NLRP3 inflammasome activation in mice. Autophagy 12 (12), 2326–2343. doi: 10.1080/15548627.2016.1235124 27780404 PMC5173275

[B18] Hannon G. J. Casso D. Beach D. (1994). KAP: a dual specificity phosphatase that interacts with cyclin-dependent kinases. Proc. Natl. Acad. Sci. U.S.A. 91 (5), 1731–1735. doi: 10.1073/pnas.91.5.1731 8127873 PMC43237

[B19] Ichinohe T. Lee H. K. Ogura Y. Flavell R. Iwasaki A. (2009). Inflammasome recognition of influenza virus is essential for adaptive immune responses. J. Exp. Med. 206 (1), 79–87. doi: 10.1084/jem.20081667 19139171 PMC2626661

[B20] Iskusnykh I. Y. Kryl'skii E. D. Brazhnikova D. A. Popova T. N. Shikhaliev K. S. Shulgin K. K.. (2021). Novel antioxidant, deethylated ethoxyquin, protects against carbon tetrachloride induced hepatotoxicity in rats by inhibiting NLRP3 inflammasome activation and apoptosis. Antioxidants (Basel) 10 (1), 122. doi: 10.3390/antiox10010122 33467773 PMC7829797

[B21] Jeong J. S. Choi J. Y. Kim J. S. Park S. O. Kim W. Yoon Y. G. (2022). SARS-CoV-2 infection in severe asthma is associated with worsening of COVID-19 through respiratory NLRP3 inflammasome activation. Allergy 78 (1), 287–290. doi: 10.1111/all.15452 35871401 PMC9349818

[B22] Jin Q. Li W. Yu W. Zeng M. Liu J. Xu P. (2022). Analysis and identification of potential type II helper T cell (Th2)-related key genes and therapeutic agents for COVID-19. Comput. Biol. Med. 150, 106134. doi: 10.1016/j.compbiomed.2022.106134 36201886 PMC9528635

[B23] Johnson W. E. Li C. Rabinovic A. (2007). Adjusting batch effects in microarray expression data using empirical Bayes methods. Biostatistics (Oxford, England) 8 (1), 118–27. 16632515 10.1093/biostatistics/kxj037

[B24] Junqueira C. Crespo Â. Ranjbar S. de Lacerda L. B. Lewandrowski M. Ingber J.. (2022). FcγR-mediated SARS-CoV-2 infection of monocytes activates inflammation. Nature 606 (7914), 576–584. doi: 10.1038/s41586-022-04702-4 35385861 PMC10071495

[B25] Lawal B. Kuo Y. C. Rachmawati Sumitra M. Wu A. T. H. Huang H. S. (2022). Identification of a novel immune-inflammatory signature of COVID-19 infections, and evaluation of pharmacokinetics and therapeutic potential of RXn-02, a novel small-molecule derivative of quinolone. Comput. Biol. Med. 148, 105814. doi: 10.1016/j.compbiomed.2022.105814 35841781 PMC9272679

[B26] Leal V. N. C. Andrade M. M. S. Teixeira F. M. E. Cambui R. A. G. Roa M. Marra L. G.. (2022). Severe COVID-19 patients show a dysregulation of the NLRP3 inflammasome in circulating neutrophils. Scand. J. Immunol. 97 (3), e13247. doi: 10.1111/sji.13247 36541819

[B27] Lenoir A. Christe A. Ebner L. Beigelman-Aubry C. Bridevaux P. O. Brutsche M.. (2022). Pulmonary recovery 12 months after non-severe and severe COVID-19: The prospective Swiss COVID-19 lung study. Respiration 102 (2), 120–133. doi: 10.1159/000528611 36566741 PMC9932828

[B28] Li X. Zhang X. Pan Y. Shi G. Ren J. Fan H.. (2018). mTOR regulates NLRP3 inflammasome activation *via* reactive oxygen species in murine lupus. Acta Biochim. Biophys. Sin. (Shanghai) 50 (9), 888–896. doi: 10.1093/abbs/gmy088 30060081

[B29] Liang W. Xie B. K. Ding P. W. Wang M. Yuan J. Cheng X.. (2021). Sacubitril/Valsartan alleviates experimental autoimmune myocarditis by inhibiting Th17 cell differentiation independently of the NLRP3 inflammasome pathway. Front. Pharmacol. 12. doi: 10.3389/fphar.2021.727838 PMC8479108 34603042

[B30] Liao M. Liu Y. Yuan J. Wen Y. Xu G. Zhao J.. (2020). Single-cell landscape of bronchoalveolar immune cells in patients with COVID-19. Nat. Med. 26 (6), 842–844. doi: 10.1038/s41591-020-0901-9 32398875

[B31] Lorente L. Martín M. M. González-Rivero A. F. Pérez-Cejas A. Argueso M. Perez A.. (2021). Blood concentrations of proapoptotic sFas and antiapoptotic Bcl2 and COVID-19 patient mortality. Expert Rev. Mol. Diagn. 21 (8), 837–844. doi: 10.1080/14737159.2021.1941880 34128765 PMC8240540

[B32] Lucas C. Wong P. Klein J. Castro T. B. R. Silva J. Sundaram M.. (2020). Longitudinal analyses reveal immunological misfiring in severe COVID-19. Nature 584 (7821), 463–469. doi: 10.1038/s41586-020-2588-y 32717743 PMC7477538

[B33] Milara J. Martínez-Expósito F. Montero P. Roger I. Bayarri M. A. Ribera P.. (2022). N-acetylcysteine reduces inflammasome activation induced by SARS-CoV-2 proteins *In vitro* . Int. J. Mol. Sci. 23 (23), 14518. doi: 10.3390/ijms232314518 36498845 PMC9738300

[B34] Mills K. H. Dungan L. S. Jones S. A. Harris J. (2013). The role of inflammasome-derived IL-1 in driving IL-17 responses. J. Leukoc. Biol. 93 (4), 489–497. doi: 10.1189/jlb.1012543 23271701

[B35] Naik R. R. Shakya A. K. Aladwan S. M. El-Tanani M. (2022). Kinase inhibitors as potential therapeutic agents in the treatment of COVID-19. Front. Pharmacol. 13. doi: 10.3389/fphar.2022.806568 PMC9014181 35444538

[B36] O'Bryan J. P. Frye R. A. Cogswell P. C. Neubauer A. Kitch B. Prokop C.. (1991). Axl, a transforming gene isolated from primary human myeloid leukemia cells, encodes a novel receptor tyrosine kinase. Mol. Cell Biol. 11 (10), 5016–5031. doi: 10.1128/mcb.11.10.5016-5031.1991 1656220 PMC361494

[B37] Ohuabunwa U. Afolabi P. Tom-Aba D. Fluker S. A. (2022). Clinical presentation of COVID-19 and association with outcomes among hospitalized older adults. J. Am. Geriatr. Soc. 71 (2), 599–608. doi: 10.1111/jgs.18163 36565152 PMC9880682

[B38] Potere N. Del Buono M. G. Caricchio R. Cremer P. C. Vecchié A. Porreca E.. (2022). Interleukin-1 and the NLRP3 inflammasome in COVID-19: Pathogenetic and therapeutic implications. EBioMedicine 85, 104299. doi: 10.1016/j.ebiom.2022.104299 36209522 PMC9536001

[B39] Sefik E. Qu R. Junqueira C. Kaffe E. Mirza H. Zhao J.. (2022). Inflammasome activation in infected macrophages drives COVID-19 pathology. Nature 606 (7914), 585–593. doi: 10.1038/s41586-022-04802-1 35483404 PMC9288243

[B40] Silvin A. Chapuis N. Dunsmore G. Goubet A. G. Dubuisson A. Derosa L.. (2020). Elevated calprotectin and abnormal myeloid cell subsets discriminate severe from mild COVID-19. Cell 182 (6), 1401–1418.e1418. doi: 10.1016/j.cell.2020.08.002 32810439 PMC7405878

[B41] Tay M. Z. Poh C. M. Rénia L. MacAry P. A. Ng L. F. P. (2020). The trinity of COVID-19: immunity, inflammation and intervention. Nat. Rev. Immunol. 20 (6), 363–374. doi: 10.1038/s41577-020-0311-8 32346093 PMC7187672

[B42] Thomas P. G. Dash P. Aldridge J. R. Jr. Ellebedy A. H. Reynolds C. Funk A. J.. (2009). The intracellular sensor NLRP3 mediates key innate and healing responses to influenza a virus *via* the regulation of caspase-1. Immunity 30 (4), 566–575. doi: 10.1016/j.immuni.2009.02.006 19362023 PMC2765464

[B43] Xu X. Wu X. Yue G. An Q. Lou J. Yang X.. (2022). The role of nod-like receptor protein 3 inflammasome activated by ion channels in multiple diseases. Mol. Cell Biochem. Advance online publication. doi: 10.1007/s11010-022-04602-1 PMC10164009 36378463

[B44] Yap J. K. Y. Moriyama M. Iwasaki A. (2020). Inflammasomes and pyroptosis as therapeutic targets for COVID-19. J. Immunol. 205 (2), 307–312. doi: 10.4049/jimmunol.2000513 32493814 PMC7343621

[B45] Yapasert R. Khaw-On P. Banjerdpongchai R. (2021). Coronavirus infection-associated cell death signaling and potential therapeutic targets. Molecules 26 (24), 7459. doi: 10.3390/molecules26247459 34946543 PMC8706825

[B46] Zdżalik-Bielecka D. Kozik K. Poświata A. Jastrzębski K. Jakubik M. Miączyńska M. (2022). Bemcentinib and gilteritinib inhibit cell growth and impair the endo-lysosomal and autophagy systems in an AXL-independent manner. Mol. Cancer Res. 20 (3), 446–455. doi: 10.1158/1541-7786.mcr-21-0444 34782372

[B47] Zhang J. Xie B. Hashimoto K. (2020). Current status of potential therapeutic candidates for the COVID-19 crisis. Brain Behav. Immun. 87, 59–73. doi: 10.1016/j.bbi.2020.04.046 32334062 PMC7175848

[B48] Zheng M. Karki R. Williams E. P. Yang D. Fitzpatrick E. Vogel P.. (2021). TLR2 senses the SARS-CoV-2 envelope protein to produce inflammatory cytokines. Nat. Immunol. 22 (7), 829–838. doi: 10.1038/s41590-021-00937-x 33963333 PMC8882317

[B49] Zhou G. Soufan O. Ewald J. Hancock R. E. W. Basu N. Xia J.. (2019). NetworkAnalyst 3.0: A visual analytics platform for comprehensive gene expression profiling and meta-analysis. Nucleic Acid Res 47 (W1), W234–W41. 30931480 10.1093/nar/gkz240 PMC6602507

