# Supplementary material for: Identifying key genes related to inflammasome in severe COVID-19 patients based on a joint model with random forest and artificial neural network
Source: Front Cell Infect Microbiol. 2023 Apr 11;13:1139998. doi: 10.3389/fcimb.2023.1139998 (PMC10126306; doi:10.3389/fcimb.2023.1139998)
Supplement: Supplementary file 1 [file DataSheet_1.docx]

Supplementary Material

**Identifying Key Genes Related to Inflammasome in Severe Patients with COVID-19 based on a Joint Model with Random Forest and Artificial Neural Network**

**Haiya Ou^1^, Yaohua Fan^2,3^, Xiaoxuan Guo^2^, Meiling Zhu^2*^, Geng Li^3*^, Lijun Zhao^2*^**

*To whom correspondence should be addressed.

***Correspondence:**  zhaoljun@mail2.sysu.edu.cn

# Supplementary Figures and Tables

## Supplementary Tables

Supplementary Table S1. 192 co-expressed DEGs related to COVID-19. (Supplementary Table S1.xls)

Supplementary Table S2. The kmeans cluster of PPI network of 192 DEGs. (Supplementary Table S2.xls)

Supplementary Table S3. The GO enrichment results of 192 DEGs. (Supplementary Table S3.xls)

Supplementary Table S4. The KEGG enrichment results of predicted genes of 192 DEGs. (Supplementary Table S4.xls)

Supplementary Table S5. 1007 inflammasome-associated genes. (Supplementary Table S5.xls)

Supplementary Table S6. The kmeans cluster of PPI network of 40 IADEGs. (Supplementary Table S6.xls)

Supplementary Table S7. The GO enrichment results of 40 IADEGs. (Supplementary Table S7.xls)

Supplementary Table S8. The KEGG enrichment results of 40 IADEGs. (Supplementary Table S8.xls)
